# 

**Published:** 1894-07

**Authors:** 


					﻿Vol. XIV.
JULY, 1894.
NO. 7.
THE
HOMEOPATHIC pHYjSlClAJfl,
A Monthly Journal of Homoeopathic Materia Medica
and Clinical Medicine.
w He m a freeman whom truth makes free, and all are slaves beside."
"Seek the Truth: come whence it may, cqgff^at it wtil."
EK M.	M.(D.
PHILADELPHIA:
ZSTo. 1231 LOCUST STREET.
lokdon:
ALFRED HEATH A CO., Bolk Agknts bob Gbbat Bbitain,
114 Ebury Street, Eaton Square.
'Yearly Subscription, $2.50, invariably in advance. Sinyle numbers, 25 cts
Enured al the Philadelphia Poav-OtBe. aa Beoood Claa. Mall Matter.
				

